# Setting Up an NGS Sequencing Platform and Monitoring Molecular Markers of Anti-Malarial Drug Resistance in Djibouti

**DOI:** 10.3390/biology13110905

**Published:** 2024-11-06

**Authors:** Nasserdine Papa Mze, Houssein Yonis Arreh, Rahma Abdi Moussa, Mahdi Bachir Elmi, Mohamed Ahmed Waiss, Mohamed Migane Abdi, Hassan Ibrahim Robleh, Samatar Kayad Guelleh, Abdoul-ilah Ahmed Abdi, Hervé Bogreau, Leonardo K. Basco, Bouh Abdi Khaireh

**Affiliations:** 1 Service de Biologie, Unité de Microbiologie, Hôpital Mignot, Centre Hospitalier de Versailles, 177 rue de Versailles, 78150 Le Chesnay, France; 2 Unité de Biologie Moléculaire, Hôpital Général de Peltier, Centre Hospitalier Universitaire de Djibouti, Avenue Marechal, Djibouti ville 98230, Djibouti; 3 Caisse Nationale de Sécurité Sociale (CNSS), Ministère de la Santé, Djibouti ville 98230, Djibouti; 4 Laboratoire du Service de Santé de la Gendarmerie Nationale, Avenue de Brazzaville, Djibouti ville 98230, Djibouti; 5 Programme National de Lutte contre le Paludisme, Ministère de la Santé, Djibouti ville 98230, Djibouti; 6 Présidence de la République de Djibouti, Commune de Ras Dika, Djibouti ville 98230, Djibouti; 7 Service de Santé des Armées, Forces Armées Djiboutiennes, Commune de Boulaos, Djibouti ville 98230, Djibouti; 8 Unité Parasitologie et Entomologie, Département Microbiologie et Maladies Infectieuses, Institut de Recherche Biomédicale des Armées, 13005 Marseille, France; 9 Centre National de Référence du Paludisme, 13005 Marseille, France; 10 IHU Méditerranée Infection, 13005 Marseille, France; 11 Aix Marseille Université, Institut de Recherche pour le Développement (IRD), Assistance Publique-Hôpitaux de Marseille (AP-HM), Service de Santé des Armées (SSA), 13005 Marseille, France; 12 Ministry of Health, Global Fund to Fight AIDS-TB-Malaria Project, Djibouti ville 98230, Djibouti

**Keywords:** Djibouti, drug resistance, malaria, next-generation sequencing, *Plasmodium falciparum*

## Abstract

Djibouti is confronted with malaria resurgence, with malaria having been occurring in epidemic proportions since a decade ago. The current epidemiology of drug-resistant *Plasmodium falciparum* is not well known. Molecular markers were analyzed by targeted sequencing in 79 *P. falciparum* clinical isolates collected in Djibouti city in 2023 using the Miseq Illumina platform newly installed in the country. The objective of the study was to analyze the key codons in these molecular markers associated with antimalarial drug resistance. The prevalence of the mutant *Pfcrt* CVIET haplotype (92%) associated with chloroquine resistance and mutant *Pfdhps-Pfdhfr* haplotypes (7.4% SGEA and 53.5% IRN, respectively) associated with sulfadoxine-pyrimethamine resistance was high. By contrast, *Pfmdr1* haplotypes associated with amodiaquine (YYY) or lumefantrine (NFD) resistance were not observed in any of the isolates. Although the “Asian-type” *PfK13* mutations associated with artemisinin resistance were not observed, the “African-type” PfK13 substitution, R622I, was found in a single isolate (1.4%) for the first time in Djibouti. Our genotyping data suggest that most Djiboutian *P. falciparum* isolates are resistant to chloroquine and sulfadoxine-pyrimethamine but are sensitive to amodiaquine, lumefantrine, and artemisinin. Nonetheless, the presence of an isolate with the R622I PfK13 substitution is a warning signal that calls for a regular surveillance of molecular markers of antimalarial drug resistance.

## Simple Summary

Malaria has claimed the lives of millions of people worldwide. In the absence of effective vaccines, treatment with malaria medicines is one of the most effective means of combating the disease. Today, we have very effective antimalarial medicines called artemisinin-based combination therapies, but the development of resistance in southeast Asian countries has become a major problem. In Africa, there are signs that malaria parasites may also become resistant to these medicines. Monitoring the parasites by studying changes in their genes (called mutations) is a practical way to follow any changes in drug resistance. Djibouti recently acquired a DNA sequencing machine that can determine the complete genetic sequence of the malaria parasite (and many other microorganisms) in a short time. We trained Djiboutian technicians and used this new machine in Djibouti to study whether the malaria parasites found in the country are resistant to medicines. We found that many parasites are resistant to the old antimalarial medicines, such as chloroquine and Fansidar, and show a few indirect signs that suggest that they can become resistant to the newer medicines. The sequencing machine will be an important asset to continue monitoring malaria parasites in Djibouti.

## 1. Introduction

Malaria remains a major public health problem worldwide. In 2021, malaria killed an estimated 619,000 people, compared with 625,000 deaths in 2020 [1]. The number of new malaria cases worldwide was estimated at 247 million. The World Health Organization (WHO) African Region continues to bear the heaviest burden of the disease, with 95% of malaria cases (approximately 234 million) and 96% of malaria deaths (approximately 593,000). Nearly 80% of malaria deaths in the African Region occurred in children under the age of five years old. In 2022, four countries in the African Region accounted for almost half of all cases worldwide: Nigeria (26.8%), the Democratic Republic of Congo (12.3%), Uganda (5.1%), and Mozambique (4.2%). In the Eastern Mediterranean region, there were seven endemic countries, accounting for 3% of malaria cases worldwide. Sudan paid the heaviest price for malaria in the region, accounting for 41% of cases, followed by Afghanistan, Djibouti, Pakistan, Somalia, and Yemen. Between 2021 and 2022, the number of malaria cases in the Eastern Mediterranean region rose sharply from 6.2 million to 8.3 million [1].

The malaria situation in the Republic of Djibouti has been evolving over the past few decades, alternating between periods of low transmission and epidemics [2]. The combination of intervention tools, such as the new artemisinin-based combination therapies (ACTs), rapid diagnostic tests (RDT) for malaria, and vector control, has brought encouraging results in the fight against malaria worldwide, in particular in Africa, including Djibouti [1]. In Djibouti, epidemics in the 1980s and 1990s gave way to residual malaria transmission in the 2000s [3]. In 2012, the Djiboutian health authorities considered malaria elimination an attainable goal [4]. However, in 2013, there was a sharp increase in the number of cases, prompting the WHO to declare a state of health emergency in the country [5]. In 2014, the country experienced a large-scale epidemic with 9439 cases [6]. The majority of cases occurred in Djibouti city and Dikhil region. All age groups of the population were affected. This malaria outbreak coincided with a reduction in funding for malaria control, leading to a decrease in the supply of RDTs and antimalarial drugs in health facilities [6]. In recent years, the level of malaria transmission in Djibouti has become hypoendemic, as in the 1970s and 2000s, with an unstable highly seasonal transmission, mainly between September and May, characterized by spatial heterogeneity from north to south [6]. Malaria transmission has been documented in both rural and urban zones. Between March and June 2019, a new malaria epidemic hit Djibouti, causing several tens of thousands of cases. Epidemiological data suggest a growing number of *Plasmodium falciparum* strains undetectable by the “classical” RDT based on the detection of *P. falciparum* histidine-rich protein 2 (PfHRP2) used in Djibouti [7,8,9]. Microscopic examination of blood smears may enable additional detection of circulating parasites in patients with negative PfHRP2-based RDT, but experienced microscopists are generally rare in many African countries. Thus, the deterioration in the malaria situation observed in Djibouti is probably only a fragmented view of the problem due to the lack of suitable diagnostic tools for malaria surveillance. In fact, the molecular tools needed to confirm malaria diagnosis and maintain the quality control of the malaria surveillance system have not been available in Djibouti [9].

During the last malaria epidemic, late treatment failures after ACT were observed in some patients, notably among French military personnel positioned in a Djiboutian military base [10]. While cases of ACT resistance have been documented in Southeast Asia since 2008 [11,12,13], their expansion into the African continent remains a hotly debated topic. To date, the molecular markers associated with artemisinin resistance in Southeast Asia, i.e., mutations in the propeller region of the *P. falciparum* Kelch protein gene on chromosome 13 (*PfK13*), have not been found in Africa [1,14,15]. In addition, a recent study suggests the emergence of novel mutations in the *PfK13* marker associated with artemisinin resistance in Africa. These mutations are distinct from those found in artemisinin-resistant Asian *P. falciparum* strains. These observations call into question the relevance of molecular surveillance targeting a single marker that is characteristic of Asian *P. falciparum* strains [16,17]. As East Africa has historically been the gateway to drug-resistant *P. falciparum* on the continent, it is urgent to document therapeutic failures after ACT in the region using suitable molecular tools.

To tackle the resurgence of malaria epidemic in Djibouti, the National Malaria Control Programme (NMCP or “Programme National de Lutte contre le Paludisme” PNLP in French) in Djibouti has updated and modified the national antimalarial drug policy and guidelines since 2013 [6]. Artemether–lumefantrine is used for first-line malaria treatment of acute uncomplicated malaria, and artesunate–amodiaquine is administered for the second-line treatment. Injectable artesunate (first line) and quinine (alternative or second line) remains the drug of choice for severe and complicated malaria. The NMCP still recommends chloroquine and primaquine for the treatment of *Plasmodium vivax* malaria. Sulfadoxine-pyrimethamine (SP) had been used for first-line treatment in the past, before the massive introduction of ACT. At present, it is reserved for intermittent preventive treatment in pregnant women (IPTp). The massive use of ACTs in the Horn of Africa (i.e., Djibouti, Somalia, Ethiopia, and Eritrea) is an important step in the fight against drug-resistant *P. falciparum* malaria. ACTs are also necessary in the continuous effort to reduce malaria-associated morbidity and mortality and reduce transmission in the sub-region. Despite considerable improvement in malaria control in the Horn of Africa, recent reports on the emergence of *P. falciparum* strains with *PfK13* mutations associated with reduced sensitivity to artemisinin are alarming [1,17,18]. While several studies have been carried out worldwide and in the Horn of Africa sub-region, very few in vitro and molecular studies on antimalarial drug resistance have been carried out in Djibouti since 2006 [3,19]. The country’s NMCP has not conducted any assessment of the therapeutic efficacy of antimalarial drugs, even though the WHO recommends evaluating therapeutic efficacy every two years [20].

It is in this context that we deemed it necessary to investigate and determine the prevalence of mutations associated with resistance to the antimalarial drugs used in Djibouti. To achieve this objective, the Djiboutian government has set up, for the first time, a genomic laboratory platform with the installation of the latest next-generation sequencer (NGS), an Illumina Miseq. The objectives of this study were to evaluate *P. falciparum* resistance markers in Djibouti using this technology.

## 2. Materials and Methods

### 2.1. Study Sites and Sample Collection

**Figure 1:**
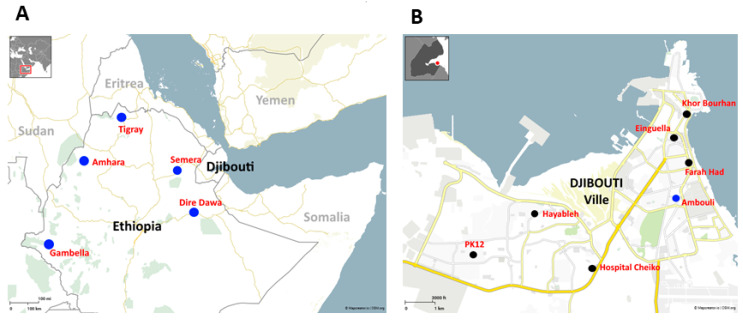
(**A**) Djibouti’s geographical position in the Horn of Africa. This map illustrates the proximity of Djibouti to Ethiopian cities where the “African-specific” R622I PfK13 amino acid substitution associated with artemisinin resistance has recently been observed, as indicated by blue dots [22,23]. (**B**) Map of Djibouti city showing the location of health centers and hospitals where blood samples from malaria-infected patients were collected. Ambouli health center, indicated by a blue dot, was the only Djiboutian study site where R622I was found in the present study. The locations of other health centers and hospitals where R622I was not observed are shown with black dots.

Djibouti is situated in the Horn of Africa (Figure 1A). This study was carried out at the molecular biology laboratory of the Centre Hospitalo-Universitaire (CHU; University Hospital Center) de Djibouti. Samples were collected from Balbala Hospital and six community health centers (Farah-Had Center, Einguella Center, Khor Bourhan Center, Ambouli Center, PK12 Center, Hayableh Center) in 2023 (Figure 1B).

Febrile patients with suspected malaria were screened for malaria using two different RDTs: the RapiGEN Biocredit malaria Ag Pf/Pv RDT (RapiGEN Inc.; Suwon, Republic of Korea; batch number H016B001D) and Bioline test (Bioline, batch number: 05EDH004B, as described in our earlier works [9,21]). RapiGEN Biocredit RDT detects two distinct lactate dehydrogenase (LDH) iso-enzymes, PfLDH, specific for *P. falciparum,* and PvLDH, specific for *P. vivax*. The Bioline test RDT detects PfHRP2 and plasmodial LDH (i.e., the genus-specific LDH common to all human *Plasmodium* spp.). The Bioline test RDT was mainly used to identify the presence of *Pfhrp2*-gene-deleted *P. falciparum* strains that do not express the PfHRP2 antigen, leading to a false negative RDT result when using a PfHRP2-based RDT. If the Biocredit RDT gave a positive result, indicating the presence of *P. falciparum*, parasite DNA was extracted from whole blood samples. A total of 79 Biocredit-RDT-positive samples were collected and used in this study.

#### 2.1.1. DNA Extraction

All samples were extracted using the Qiagen QIAcube robot (QIAcube HT, San Francisco, CA, USA) and Qiagen whole blood extraction protocol, as recommended by the manufacturer. For each sample, 200 µL of blood was extracted to obtain a final volume of 200 µL of eluent.

#### 2.1.2. DNA Amplification

All 79 samples extracted in this study were amplified for the *PfK13* gene. Of these, 31 were selected for the genotyping five additional molecular markers associated with drug resistance: *P. falciparum* chloroquine resistance transporter (*Pfcrt*), associated with chloroquine resistance; *P. falciparum* multidrug resistance 1 (*Pfmdr1*), associated with resistance to chloroquine, amodiaquine, and lumefantrine; *P. falciparum* dihydrofolate reductase (*Pfdhfr*), associated with pyrimethamine resistance; and *P. falciparum* dihydropteroate synthase (*Pfdhps*), associated with sulfadoxine resistance.

For the *PfK13* gene, the polymerase chain reaction (PCR) mixture consisted of 3 μL of DNA template, 10 μL of PCR master mix, 0.25 μL of 100 μM primers, and 6.40 μL of nuclease-free water. The thermal cycler was programmed as follows: initial denaturation of 95 °C for 15 min, followed by 45 cycles of 95 °C for 30 min, 60 °C for 1 min, and 72° for 5 min, then a final extension at 72 °C for 5 min.

For the *Pfcrt* gene, the reaction mixture consisted of 5 μL of DNA template, 10 μL of PCR master mix, 0.2 μL of 100 μM primers, and 3 μL of nuclease-free water. The program of thermal cycling was as follows: initial denaturation of 95 °C for 5 min, followed by 40 cycles of 95 °C for 30 min, 44 °C for 20 s, and 60 °C for 1 min, then a final extension at 60 °C for 30 min.

For the *Pfmdr1* gene, two overlapping fragments were amplified due to the large size of the gene and the distance separating the key codons associated with drug resistance. The reaction mixture consisted of 5 μL of DNA template, 10 μL of PCR master mix, 0.2 μL of 100 μM primers, and 6 μL of nuclease-free water. The PCR program was as follows: initial denaturation at 95 °C for 5 min, followed by 40 cycles of 95 °C for 30 min, 64 °C for 2 min, and 72 °C for 3 min, then a final extension at 60 °C for 30 min.

For the *Pfdhfr* gene, the PCR mixture consisted of 2 μL of DNA template, 10 μL of PCR master mix, 0.2 μL of primers at 100 μM, and 6 μL of nuclease-free water. The PCR program was as follows: initial denaturation at 94 °C for 5 min, followed by 45 cycles of 94 °C for 20 s, 49 °C for 20 sec, and 72 °C for 1 min, then a final extension at 72 °C for 2 min.

For the *Pfdhps* gene, the reaction mixture consisted of 4 μL of DNA template, 10 μL of PCR master mix, 0.2 μL of 100 μM primers, and 4 μL of nuclease-free water. The thermal cycler was programmed as follows: initial denaturation of 94 °C for 3 min, followed by 45 cycles of 94 °C for 1 s, 51 °C for 20 s, and 72 °C for 1 min, then a final extension at 72 °C for 30 min.

The primer sequences and sizes are shown in Table 1. For each PCR amplification, a positive and a negative control were used. At the end of each PCR run, the PCR product was diluted 1/10 before sequencing.

#### 2.1.3. Sequencing Library Preparation

Targeted sequencing was performed in Djibouti city under the training program sponsored by the French government agency, Expertise France (Paris, France). The Miseq Illumina sequencer was provided to the Djiboutian government as part of the Global Fund grant awarded to the Djiboutian government to reinforce its technical capacity to control malaria, tuberculosis, and human immunodeficiency virus (HIV).

The library was prepared using Illumina’s Nextra XT kit (Illumina, Inc.; San Diego, CA, USA). The first step involved tagging with 5 µL of amplified DNA mixed with 10 μL of Tagment DNA Buffer and Amplicon Tagment Mix. The mixture was incubated at 55 °C for 10 min. Tagmentation was stopped with 5 µL of Neutralize Tagment solution by incubating at room temperature for 5 min. The second step involved PCR followed by purification of the libraries. The fragment product was mixed with 15 μL of Nextera PCR Master Mix (Illumina) and 10 μL of index (5 µL of index I5 and 5 µL of index I7). PCR was performed under the following conditions: initialization at 72 °C for 3 min, initial denaturation at 98 °C for 3 min, followed by 12 denaturation cycles at 98 °C for 20 s, annealing at 65 °C for 5 min, extension at 72 °C for 2 min, and final extension step at 72 °C for 5 min. After PCR, the amplified products were purified using 40 µL of AMPures beads and 200 µL of 80% ethanol. DNA fragments were eluted with 50 µL of Resuspension Buffer. The final step was to normalize the libraries using Library Normalization Beads 1, followed by elution using 30 µL of 0.1N NaOH mixed with 30 µL of Library Normalization Storage Buffer 1. This step was followed by taking 5 µL of each sample to form a pooled mixture. The pooled mixture (24 µL) was added to 576 µL of Hybridization Buffer for denaturation at 96 °C for 5 min. The denatured library was loaded into the Miseq Illumina sequencer using a 500-cycle flow cell v2 (MiSeq Reagent Kit V2).

#### 2.1.4. Data Analysis

Sequence alignment was performed using minimap2 [24] and samtools (v. 1.13) [25]. The sam files from the mapping were sorted and converted to bam. Using sam2consensus, a consensus sequence of genomes in fasta file format was generated. All fasta sequences were visualized using Geneious Prime 2024.0.2 (Biomatters, Inc., Boston, MA, USA) [26]. To confirm the presence of new and rare mutations, bam files were indexed using samtools for viewing in Integrative Genomics Viewer (version 2.16.0) [27]. The prevalence of mutant parasites was determined by calculating the number of parasites carrying a mutant allele or mutant haplotype divided by the total number of parasites (i.e., wild-type and mutant) with interpretable sequencing data for a given molecular marker.

To compare the results obtained in this study with those of 2005 [3], Fisher’s exact test was used, with the significance level set at *p* < 0.05.

## 3. Results

### 3.1. Mutant Alleles Associated with Drug Resistance

Targeted sequencing was performed to characterize the molecular markers of resistance in 79 *P. falciparum* clinical isolates in this study. Of the 79 isolates, 52.7% (39/74) lacked the *Pfhrp2* gene. There was no association (*p* > 0.05) between the absence of the *Pfhrp2* gene and the other *P. falciparum* resistance marker genes analyzed in this study.

For *PfK13*, 68 of 79 (86%) samples were correctly sequenced for codon 662 (87.3%, 69/79 for codon 189 and 86.1%, 68/79 for codon 281). Of 31 samples sequenced for *Pfcrt*, *Pfmdr1*, *Pfdhfr*, and *Pfdhps*, 80% (25/31) were correctly sequenced for *Pfcrt*, 87% (27/31) for *Pfmdr1*, 90.3% (28/31) for *Pfdhfr*, and 87% (27/31) for the *Pfdhps* gene.

In the *PfK13* gene, mutations were found at codons K189C (17.4%, 12/69), R281N (10.3%, 7/68), and R622I (1.4%, 1/68) (Table 2).

A high prevalence of mutant alleles was found in the *Pfcrt* gene, specifically at codons M74I and N75E, with 92% (23/25) at both of these codons and 96% (24/25) at codon K76T. Only 4% (1/25) of samples had the C72S mutation.

In the *Pfmdr1* gene, we found a new non-synonymous mutation at codon K189 in one isolate (3.7%, 1/27). A high prevalence of mutation was observed at codon 184 (96.3%, 26/27). No mutations were found in codons 86 and 1246.

In the *Pfdhfr* gene, high prevalences of mutations were found at codons N51I and S108N, with 82.1% (23/28) at both codons, while for codon C59R, the prevalence of a mutant allele was slightly lower (57.1%, 16/28).

For the *Pfdhps* gene, there were more *P. falciparum* isolates with mutations at codon K540E, with 77.8% (21/27), compared with codon G437A, with 29.6% (8/27) mutant allele.

### 3.2. Haplotypes Associated with Drug Resistance

Data analysis by haplotype is presented in Table 3. In the *PfK13* gene, the mutant CNCR double-mutant haplotype was present in 8.8% of the isolates. Several double-mutant *Pfcrt* haplotypes, CVIEK, CVINT, and CVMET, and the triple mutant haplotype CVIET were found at a high prevalence (92%). The latter triple mutant haplotype (i.e., CVIET) was associated with the presence of a *Pfmdr1* Y184F mutation (*p* = 0.04). The analysis of the *Pfmdr1* haplotype based on codons 86, 184, and 1246 showed that the haplotypes associated with amodiaquine resistance (YYY) and lumefantrine resistance (NFD) in Africa were absent in our isolates.

In the *Pfdhfr* gene, the double-mutant haplotypes ICNI and IRSI showed high prevalences (53.5%) compared with the wild-type haplotype NYSND. No mutations were observed in the IRNL quadruple-mutant haplotype. In the *Pfdhps* gene, only the double-mutant SGEA haplotype was observed.

Several isolates displayed multiple mutations in several genes associated with drug resistance, ranging from quadruple to nonuple mutations. Twelve isolates were characterized to be the NEFIET sextuple-mutant haplotype, a prevalence of 57.2%. Eleven had the INEFIET septuple-mutant haplotype, a prevalence of 52.3%. Only one isolate had the NINEFIET octuple-mutant haplotype. Two additional isolates displayed the IRNAERIET nonuple-mutant haplotype, with prevalences of 6.2% and 12.5%, respectively.

### 3.3. PfK13 R622I Allele and Pfhrp2 Deletion

The single isolate with an R622I substitution in PfK13 was obtained from a 9-year-old boy (code number Djib_016) who presented spontaneously for medical consultation at Ambouli health center at the beginning of April 2023. Interestingly, this patient was infected with *P. falciparum* parasites that harbored the IIRNNAFIEF nonuple-mutant haplotype and with a deleted *Pfhrp2* gene (Table 4). Other patients from Ambouli health center were infected with *P. falciparum* strains with the IIRNNAFIEF nonuple-mutant haplotype.

### 3.4. Study Sites and the Presence of Mutant Alleles

Investigation of the regions studied in this study showed that 100% of isolates from Ambouli health center had mutations in the *Pfdhfr* gene (at codons N51I and S108N) and the *Pfcrt* gene (at codons M74I, N75E, and K76T). The IIRNNAFIEF haplotype was found in this region at the prevalence of 33.3%. In the *P. falciparum* isolates collected in Eingula health center, the prevalence of the mutant alleles *Pfdhps* K540E, *Pfmdr1* Y184F, and *Pfcrt* M74I, N75E, and K76T were found to be 100%. The IRNAERIET haplotype was found at a prevalence of 33.3% in malaria-infected patients consulting this health center. In the isolates collected at Farah health center, there were 100% mutations in the *Pfdhfr* N51I/S108N alleles, the *Pfmdr1* Y184F allele, and the *Pfcrt* M74I/N75E/K76T alleles. The octuple NINEFIET haplotype was found in this center at a prevalence of 50%.

### 3.5. Evolution of the Prevalence Rates of Mutant Alleles in Djibouti City

**Figure 2:**
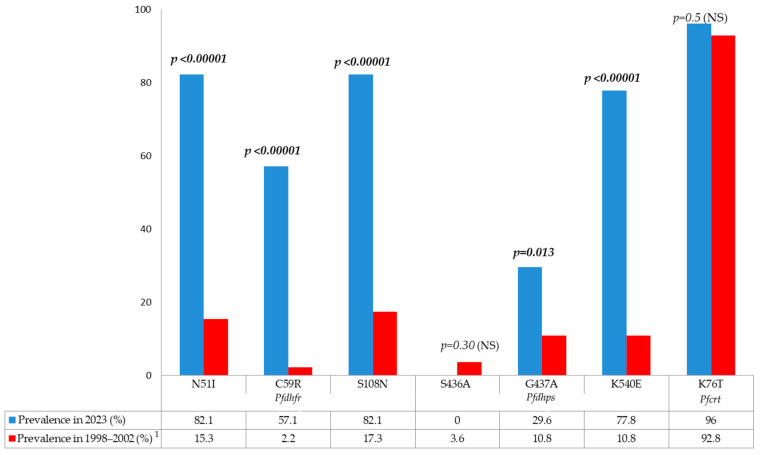
Comparison of the prevalence of mutant alleles in the molecular markers between Djiboutian isolates collected in 1998–2002 or 2023. ^1^ In the study conducted by Rogier et al. [3], *P. falciparum* isolates were collected at different periods from 1998 to 2002 (a total of 139 samples). A few isolates presented mixed alleles (i.e., both wild-type and mutant alleles). Mixed isolates were not taken into consideration to calculate the prevalence of mutant isolates. NS, not significant; *Pfdhfr*, *P. falciparum* dihydrofolate reductase; *Pfdhps*, *P. falciparum* dihydropteroate synthase; *Pfcrt*, *P. falciparum* chloroquine resistance transporter.

Our results were compared to those of Rogier et al. [3], who collected and analyzed *P. falciparum* isolates from several health centers and hospitals in Djibouti city during different time periods between 1998 and 2002 (Figure 2). Based on the assumption that the results are comparable (i.e., same study sites and similar patient inclusion criteria), our results suggest a significant increase in the prevalence of antifolate-resistant *P. falciparum* isolates, with a notable increase in the proportion of triple *Pfdhfr* mutations (based on codons 51, 59, and 108) and a marked increase in the proportion of isolates carrying the *Pfdhps* K540E mutation. By contrast, chloroquine-resistant *P. falciparum* had already reached a fixation point, with over 90% of isolates presenting the key mutant codon K76T back in the 1990s and early 2000s.

## 4. Discussion

Despite several programs set up over 60 years ago to eradicate malaria worldwide, this disease still poses a public health problem throughout the world. In 2020, the annual number of reported malaria cases in Djibouti stood at 73,535, with 19 deaths [28]. The number of malaria cases decreased to 38,877 in 2023, although this may not reflect an accurate count of malaria cases due to the negative impact that coronavirus disease 2019 (COVID-19) had on many health systems in Africa. At present, the country is still continuing its efforts to strengthen and maintain health services after the devastating impact of the COVID-19 pandemic. In this respect, Djibouti has sufficient funds to procure the necessary doses of ACTs for the coming years. The country has been monitoring insecticide resistance since 2015 and has reported the results to the WHO. The Djiboutian health authorities are planning to nominate a council and inject funds for the elimination of malaria from the country. It is in this context that the setting up of a genomic sequencing platform has become a major preoccupation for molecular epidemiological surveillance and for the reduction of malaria cases in the country. In collaboration with the Global Fund, which has agreed to support Djibouti in the fight against malaria, tuberculosis, and HIV, this international organization has granted Djibouti a state-of-the-art sequencer, an Illumina Miseq. The French government, via Expertise France, sent an international expert to train Peltier laboratory technicians from the University Hospital Center in Djibouti in genomic sequencing, with particular emphasis on *P. falciparum*. During this training session, the molecular markers of drug-resistant *P. falciparum* were studied.

After two months of successful training, the sequencing results showed a high prevalence of mutations associated with antimalarial drug resistance in the *Pfcrt*, *Pfmdr1*, *Pfdhfr*, and *Pfdhps* genes. If we compare the results obtained in this study with those obtained by Rogier et al. in isolates collected during the period 1998–2002 [3], we find a significant increase in the number of mutations in the *Pfdhfr* N51I alleles (15.1% in 1998–2002 vs. 82.1% in 2023; *p* < 0.00001), C59R (2.2% in 1998–2002 vs. 57.1% in 2023; *p* < 0.00001), and S108N (17.3% in 1998–2002 vs. 82.1% in 2013; *p* < 0.00001). Similar findings were observed in the *Pfdhps* alleles G437A (10.8% in 1998–2002 vs. 29.6% in 2023) and K540E (10.8% in 1998–2002 vs. 77.8% in 2023) (Figure 2). This sharp increase in the prevalence of mutant alleles in these two genes may be due to self-medication with sulfadoxine-pyrimethamine (Fansidar^®^) or the use of sulfamethoxazole-trimethoprim (Bactrim^®^) [29,30]. The latter drug is similar to SP and has the ability to select antifolate-resistant mutants. Despite the high prevalence of key mutations in *Pfdhfr-Pfdhps*, the WHO still recommends SP for IPTp in African countries because there is currently insufficient clinical evidence that these mutant isolates compromise the efficacy of IPTp based on SP administration [20,31]. The situation is different when SP is used for treatment in combination with artesunate. For example, in Somalia, where the prevalence of *Pfdhfr-Pfdhps* mutations is high, the use of artesunate-SP as a first-line treatment may result in clinical failure in >10% of patients [32,33]. In contrast to the situations in Djibouti and Somalia, our results differ from those obtained in Ethiopia, where high prevalence of mutant *Pfdhfr* and *Pfdhps* alleles (54–90%) was reported in the 2000s [34,35,36], followed by a decrease in the prevalence of combined *Pfdhfr-Pfdhps* mutations, especially after the withdrawal of SP for treatment [37,38]. This tendency in Ethiopia requires further molecular analysis in more recent *P. falciparum* isolates collected in the country.

In the *Pfcrt* gene, the high prevalence of mutations found in this study was similar to that reported earlier by Rogier et al. [3] (92% in 1998–2002 and 96% in 2023; *p* = 0.5). Even after close to two decades following the discontinuation of the use of chloroquine for *P. falciparum* infections, the prevalence of *Pfcrt* mutations remains unchanged and is close to fixation. Similar observations were reported from Ethiopia, where the prevalence of the K76T mutant allele was 91.6% in a recent study [39]. On the other hand, our results are very different from those observed in West and Central Africa, where the prevalence of mutant *Pfcrt* has considerably decreased over the years in some countries [40,41,42]. This phenomenon has led some West and Central African countries to consider a return to the use of chloroquine [43,44,45,46]. At present, however, ACTs are still highly effective in treating multidrug-resistant *P. falciparum* in Africa. The use of chloroquine monotherapy to treat *P. falciparum* malaria is not justified outside strictly controlled clinical trials. We recall that, in a few African countries where *P. vivax* is endemic, chloroquine is usually recommended unless clinical studies have shown the presence of chloroquine-resistant *P. vivax* strains [20]. In Djibouti, chloroquine is currently used in combination with primaquine for the treatment of *P. vivax*, the prevalence of which seems to be increasing in the country [9].

In the present study, we found a very high prevalence of 92.6% for the *Pfmdr1* Y184F mutant allele. This result may be of concern, as this mutation, in combination with the wild-type allele N86 and the mutant allele 1246D, is associated with resistance to lumefantrine, the current first-line treatment in Djibouti. The clinical efficacy of artemether–lumefantrine is still very high, and the NFD *Pfmdr1* haplotype (based on codons 86, 184, and 1246) was not found in our study. If further molecular surveillance encounters an increasing prevalence of NFD-type *P. falciparum* strains, replacing lumefantrine with amodiaquine would be advantageous. We did not find the N86Y mutation, and there is positive selection of the *Pfcrt* 76T and *Pfmdr1* 86Y mutant alleles exerted by amodiaquine [47,48]. Moreover, the in vitro study carried out by Pradines et al. showed that there was no amodiaquine-resistant Djiboutian *P. falciparum* among the isolates collected in 1999 [19]. The high prevalence of the Y184F *Pfmdr1* amino acid substitution found in this study is not in agreement with the reports from other countries in the sub-region. In Ethiopia and Kenya, the prevalence of *P. falciparum* isolates carrying this mutation was low [45,49]. Interestingly, a new *Pfmdr1* synonymous mutation in codon G182 was observed in a single sample in our study. This is the first time that this mutation has been observed anywhere in the world. Other Djiboutian *P. falciparum* isolates are known to carry this synonymous mutation, but its possible impact on drug resistance is still unknown (personal communication, R. Abdi Moussa, unpublished data).

Our analysis of the *PfK13* gene showed the presence of the R622I mutation at a very low prevalence. This mutation causes partial resistance to artemisinin [50,51]. This is the first time that the R622I mutation has been observed in Djibouti. This mutation has been observed in Somalia, Sudan, Mozambique, Zambia, and Eritrea in recent years [52,53]. This *PfK13* mutation has also been reported recently in Ethiopia [22,23] and confirmed by the WHO. These data suggest a rapid expansion of *P. falciparum* strains carrying the R622I mutation in the sub-region. The spread of *P. falciparum* strains with an R622I mutation has become a major concern in Africa, as this may compromise the efficacy and use of artemisinin derivatives. Therefore, an increased molecular and clinical surveillance of the spread of artemisinin-resistant *P. falciparum* parasites is required to arrest its rapid expansion in Africa.

The PfK13 R622I substitution was observed in the Ethiopian towns of Dire Dawa and Semera [23] (see Figure 1A). Due to the proximity of Dire Dawa to the Ethiopian–Djiboutian border and the economic and social ties between Djibouti and Dire Dawa, it is possible that Djiboutians visiting Dire Dawa in Ethiopia, especially during the summer period when malaria transmission reaches the peak season (September to December following the rainy season) in Ethiopia, may be infected in Ethiopia and import malaria strains when they return to their home country. Moreover, commercial goods transit through Dire Dawa on the way to the port of Djibouti, which may also represent a route of expansion for drug-resistant plasmodial strains.

In addition to the R622I substitution in PfK13, we found, for the first time in the Horn of Africa, the novel *PfK13* mutations K189C and R281N, which have been recently observed in sub-Saharan Africa and Cameroon, respectively [54,55]. At present, other than ensuring treatment compliance, good drug quality, close follow-up in case of treatment failure, and molecular monitoring of *PfK13*, there are no specific recommended measures to be taken when confronted with *P. falciparum* strains carrying R622I in Africa. Although ACT is still effective in Africa, the presence of these mutations in Djiboutian *P. falciparum* clinical isolates is alarming. Djibouti needs to intensify malaria surveillance along its borders, and countries in the Horn of Africa need a common, sub-regional strategy to face the challenge of the possible emergence of artemisinin-resistant malaria.

There are several limitations in the present study. Given the fact that this study was part of the first training session of Djiboutian technicians in the use of NGS, the number of samples available for analysis was relatively small. The samples were collected from different health centers and hospitals in Djibouti city. Further studies including a large number of samples from other areas of the country are required to map the current distribution of drug-resistant *P. falciparum* malaria in the country. Moreover, similar molecular analysis of Djiboutian *P. vivax* isolates would be of vital interest to monitor drug resistance in this malaria species, the incidence of which seems to be increasing in the country. More cooperative efforts in capacity building and training are required to overcome other technical limitations and go beyond molecular tools, such as in vitro drug sensitivity assays and the clinical evaluation of drug efficacy, in Djibouti.

## 5. Conclusions

This study showed the presence of drug-resistant Djiboutian isolates of *P. falciparum* on the basis of the sequences of molecular markers determined by NGS and the high prevalence of mutant alleles in the *Pfcrt*, *Pfmdr1*, *Pfdhfr*, and *Pfdhps* genes. Although these molecular data suggest the presence of clinical isolates of *P. falciparum* with genotypes associated with antimalarial drug resistance, especially against antifolate sulfadoxine-pyrimethamine and 4-aminoquinoline (chloroquine); these data need to be confirmed in therapeutic efficacy studies, which is the reference method to evaluate antimalarial drug resistance. At present, sulfadoxine-pyrimethamine and artemether–lumefantrine are still effective for IPTp and first-line treatment of acute uncomplicated *P. falciparum* malaria, respectively. If the proportion of isolates with the *Pfmdr1* haplotype NFD increases, accompanied by artemether–lumefantrine treatment failures, the first-line use of artesunate–amodiaquine may be warranted. The observation of the R622I substitution in PfK13 is a wake-up call for the country. As an immediate response, molecular surveillance for antimalarial drug-resistant *P. falciparum* strains should be reinforced, not only in Djibouti but also throughout the sub-region. Our study showed that Djiboutian researchers and technicians are now in a position to carry out genomic studies using NGS, which may also be applied and extended to assess molecular markers of resistance in *P. vivax* and initiate molecular studies on the malaria vector.

## Figures and Tables

**Table 1:** Primers used to amplify and sequence molecular markers of antimalarial drug resistance.

Gene (Chromosome ^1^)	Forward and Reverse Primer Sequences (5′-3′) ^2^	Size (bp)	Key Mutations ^3^
*PfK13* (13)	GCCAAGCTGCCATTCATTTG	849	C580Y (causal)
	GCCTTGTTGAAAGAAGCAGA		
*Pfcrt* (7)	GTTCTTGTCTTGGTAAATGT	148	K76T (causal)
	CCAATTTTGTTTAAAGTTCT		
*Pfmdr1* (5) fragment A	GTGCTGTATTATCAGGAGGAACA	423	N86Y, Y184F, S1034C, N1042D, D1246Y (associated)
	ACGGAAAAACGCAAGTAATACA		
*Pfmdr1* (5) fragment B	GTCAAGCGGAGTTTTTGCAT	973	
	AGCAGCAAACTTACTAACACG		
*Pfdhfr* (4)	ACGTTTTCGATATTTATGC	562	S108N, N51I, C59R, I164L (causal)
	TCACATTCATATGTACTATTTATTC		
*Pfdhps* (8)	GTTGAACCTAAACGTGCTGT	672	A437G + K540E (causal)
	TTCATCATGTAATTTTTGTTGTG		

^1^ There are 14 chromosomes in *P. falciparum*. The chromosome number is given in parentheses. ^2^ For each molecular marker, the forward and reverse primers are given in this order. ^3^ The key mutation and its degree of association with drug resistance (causal or associated) are presented. For PfK13, C580Y is the key codon associated with artemisinin resistance in Southeast Asia. At present, C580Y is not considered to be the key codon in African *P. falciparum* strains. bp, base pairs.

**Table 2:** Prevalence of point mutations found in Djiboutian *P. falciparum* isolates.

Gene	Codon	Allele	Genotype	Prevalence *n* (%)
*PfK13*	K189C	K189	Wild type	57 (82.6)
		189**C**	Mutant	12 (17.4)
	R281N	R281	Wild type	61 (89.7)
		281N	Mutant	7 (10.3)
	R662I	R622	Wild type	67 (98.6)
		622I	Mutant	1 (1.4)
*Pfcrt*	C72S	C72	Wild type	24 (96)
		72S	Mutant	1 (4)
	V73K	V73	Wild type	25 (100)
		73K	Mutant	0 (0)
	M74I	M74	Wild type	2 (8)
		74I	Mutant	23 (92)
	N75E	N75	Wild type	2 (8)
		75E	Mutant	23 (92)
	K76T	K76	Wild type	1 (4)
		76T	Mutant	24 (96)
*Pfmdr1*	N86Y	N86	Wild type	27 (100)
		86Y	Mutant	0 (0)
	G182G *	G182	Wild type	26 (96.3)
		182G	Mutant	1 (3.7)
	Y184F	Y184	Wild type	1 (3.7)
		184F	Mutant	26 (96.3)
	D1246Y	D1246	Wild type	27 (100)
		1246Y	Mutant	0 (0)
*Pfdhfr*	N51I	N51	Wild type	5 (17.9)
		51I	Mutant	23 (82.1)
	C59R	C59	Wild type	12 (42.9)
		59R	Mutant	16 (57.1)
	S108N	S108	Wild type	5 (17.9)
		108N	Mutant	23 (82.1)
*Pfdhps*	G437A	G437	Wild type	19 (70.4)
		437A	Mutant	8 (29.6)
	K540E	K540	Wild type	6 (22.2)
		540E	Mutant	21 (77.8)

* Synonymous mutation. *n*, number of isolates.

**Table 3:** Prevalence of mutant haplotypes observed in Djiboutian *P. falciparum* isolates.

Gene and Codons	Haplotype	Genotype	Prevalence *n* (%)
*PfK13* (189-281-580-622)	KRCR	Wild type	54 (79.4)
	**CN**CR	Double mutant	6 (8.8)
	**C**RC**I**	Double mutant	0 (0)
	K**N**C**I**	Double mutant	0 (0)
	**CNYI**	Quadruple mutant	0 (0)
*Pfcrt* (72-73-74-75-76)	CVMNK	Wild type	1 (4)
	**S**VMN**T**	Double mutant	1 (4)
	CV**IE**K	Double mutant	23 (92)
	CV**I**N**T**	Double mutant	23 (92)
	CVM**ET**	Double mutant	23 (92)
	CV**IET**	Triple mutant	23 (92)
*Pfmdr1* (86-184-1034-1042-1246) ^1^	NYSND	Wild type	1 (3.7)
	**YF**SND	Double mutant	0 (0)
	**YY**SN**Y**	Triple mutant	0 (0)
*Pfdhfr* (51-59-108-164)	NCSI	Wild type	4 (14.3)
	IRSI	Double mutant	15 (53.5)
	ICNI	Double mutant	16 (57.1)
	IRNI	Triple mutant	15 (53.5)
	IRNL	Quadruple mutant	0 (0)
*Pfdhps* (436-437- 540-581)	SAKA	Wild type	0 (0)
	AGKA	Double mutant	0 (0)
	SGEA	Double mutant	2 (7.4)
	SGEG	Triple mutant	0 (0)
	AGEG	Quadruple mutant	0 (0)
*Pfdhfr-Pfdhps* (51-59-108-164- 436-437-540-581)	NCSISGKA	Wild type	0 (0)
	**I**CSI**A**KA	Double mutant	7 (29.1.3)
	**I**CSIG**E**A	Double mutant	15 (62.5)
	N**R**SI**A**KA	Double mutant	7 (29.1)
	N**R**SIG**E**A	Double mutant	9 (37.5)
	NC**N**IS**AE**A	Triple mutant	2 (8.3)
	**IRN**IS**A**KA	Quadruple mutant	7 (29.1)
	**IRN**IS**AE**A	Quintuple mutant	2 (8.3)
*Pfdhfr-Pfdhps-Pfmdr1-Pfcrt* (108-540-184-74-75-76)	NEFIET	Sextuple mutant	12 (57.1)
*Pfdhfr-Pfmdr1-Pfcrt* (51-59-184-74-75-76)	IRFIET	Septuple mutant	10 (47.6)
*Pfdhfr-Pfmdr1-Pfcrt* (51-59-108-74-75-76)	IRNIET	Septuple mutant	10 (47.4)
*Pfdhfr-Pfdhps-Pfmdr1-Pfcrt* (51-108-540-184-74-75-76)	INEFIET	Septuple mutant	11 (52.3)
*PfK13-Pfdhfr-Pfdhps-Pfmdr1-Pfcrt* (281-51-108-540-184-74-75-76)	NINEFIET	Octuple mutant	1 (8.3)
*Pfk13-Pfdhfr-Pfdhps-Pfmdr1-Pfcrt* (622-51-59-108-437-184-74-75-76)	IIRNNAFIEF	Nonuple mutant	1 (6.2)
*Pfdhfr-Pfdhps-Pfmdr1-Pfcrt* (51-59-108-437-540-184-74-75-76)	IRNAERIET	Nonuple mutant	2 (9.5)

^1^ The *Pfmdr1* haplotypes (codons 86, 184, and 1246) YYY, associated with amodiaquine resistance, and NFD, associated with lumefantrine resistance, in African *P. falciparum* isolates were absent. Mutant alleles are highlighted in bold characters.

**Table 4:** Genotype of 18 *Pfhrp2*-deleted Djiboutian *P. falciparum* isolates.

Isolate Code	*PfK13*	*Pfdhfr*	*Pfdhps*	*Pfmdr1*	*Pfcrt*
Djib_001	ND	51I/C59/108N	ND	ND	ND
Djib_002	K189/D281/R622	51I/C59/108N	G437/540E	N86/184F/D1246	72S/M74/N75/76T
Djib_004	K189/281N/R622	51I/C59/108N	G437/540E	N86/184F/D1246	C72/74I/75E/76T
Djib_006	K189/D281/R622	N51/C59/S108	G437/540E	N86/184F/D1246	C72/74I/75E/76T
Djib_007	K189/D281/R622	51I/59R/108N	G437/540E	N86/184F/D1246	C72/74I/75E/76T
Djib_008	K189/D281/R622	N51/C59/S108	ND	ND	ND
Djib_012	ND	51I/59R/108N	G437/540E	N86/184F/D1246	C72/74I/75E/76T
Djib_013	K189/D281/R622	N51/C59/S108	G437/540E	N86/184F/D1246	C72/74I/75E/76T
Djib_014	ND	N51/C59/S108	G437/540E	N86/184F/D1246	C72/74I/75E/76T
Djib_015	ND	N51/C59/S108	G437/540E	N86/184F/D1246	C72/74I/75E/76T
Djib_016	K189/D281/622I	51I/59R/108N	437A/K540	N86/184F/D1246	C72/74I/75E/76T
Djib_017	K189/D281/R622	N51/C59/S108	G437/540E	N86/184F/D1246	C72/74I/75E/76T
Djib_019	K189/D281/R622	51I/59R/108N	G437/540E	N86/184F/D1246	C72/74I/75E/76T
Djib_020	ND	51I/59R/108N	437A/K540	N86/184F/D1246	C72/74I/75E/76T
Djib_021	K189/D281/R622	N51/C59/S108	437A/K540	N86/184F/D1246	C72/74I/75E/76T
Djib_023	K189/D281/R622	51I/C59/S108	437A/540E	N86/184F/D1246	C72/74I/75E/76T
Djib_025	ND	51I/59R/108N	437A/K540	N86/184F/D1246	ND
Djib_031	K189/D281/R622	51I/59R/108N	437A/540E	N86/184F/D1246	C72/74I/75E/76T

ND, not determined.

## Data Availability

The original contributions presented in this study are included in the article. Further inquiries can be directed to the corresponding author.

## Abbreviations

ACT, artemisinin-based combination therapy; bp, base pairs; COVID-19, coronavirus disease 2019; HIV, human immunodeficiency virus; IPTp, intermittent preventive treatment in pregnant women; LDH, lactate dehydrogenase; NGS, next-generation sequencing (or sequencer); NMCP, National Malaria Control Programme; PCR, polymerase chain reaction; PfHRP-2, *P. falciparum* histidine-rich protein 2; *Pfcrt*, *P. falciparum* chloroquine resistance transporter; *Pfdhfr*, *P. falciparum* dihydrofolate reductase gene; *Pfdhps*, *P. falciparum* dihydropteroate synthase gene; *PfK13*, gene coding for *P. falciparum* Kelch protein; PfLDH, lactate dehydrogenase specific for *P. falciparum*; *Pfmdr1*, *P. falciparum* multidrug resistance 1 gene; PNLP, Programme National de Lutte contre le Paludisme (Malaria Control Unit); PvLDH, lactate dehydrogenase specific for *P. vivax*; RDT, rapid diagnostic test; SP, sulfadoxine-pyrimethamine; WHO, World Health Organization.
